# Rectal Swabs as an Alternative Sample Collection Method to Bulk Stool for the Real-Time PCR Detection of *Giardia duodenalis*

**DOI:** 10.4269/ajtmh.19-0909

**Published:** 2020-06-08

**Authors:** Jacqueline R. M. A. Maasch, Ahmed M. Arzika, Catherine Cook, Elodie Lebas, Nils Pilotte, Jessica R. Grant, Steven A. Williams, Jeremy D. Keenan, Thomas M. Lietman, Kristen Aiemjoy

**Affiliations:** 1 Department of Biological Sciences, Smith College, Northampton, Massachusetts;; 2 The Carter Center, Niamey, République du Niger;; 3 Proctor Foundation, University of California San Francisco, San Francisco, California;; 4 Molecular and Cellular Biology Program, University of Massachusetts, Amherst, Massachusetts;; 5 Division of Infectious Diseases and Geographic Medicine, Department of Medicine, Stanford University School of Medicine, Stanford, California

## Abstract

Though bulk stool remains the gold standard specimen type for enteropathogen diagnosis, rectal swabs may offer comparable sensitivity with greater ease of collection for select pathogens. This study sought to evaluate the validity and reproducibility of rectal swabs as a sample collection method for the molecular diagnosis of *Giardia duodenalis.* Paired rectal swab and bulk stool samples were collected from 86 children ages 0–4 years living in southwest Niger, with duplicate samples collected among a subset of 50 children. Infection was detected using a previously validated real-time PCR diagnostic targeting the small subunit ribosomal RNA gene. *Giardia duodenalis* was detected in 65.5% (55/84) of bulk stool samples and 44.0% (37/84) of swab samples. The kappa evaluating test agreement was 0.81 (95% CI: 0.54–1.00) among duplicate stool samples (*N* = 49) and 0.75 (95% CI: 0.47–1.00) among duplicate rectal swabs (*N* = 48). Diagnostic sensitivity was 93% (95% CI: 84–98) by bulk stool and 63% (95% CI: 49–75) by rectal swabs. When restricting to the lowest three quartiles of bulk stool quantitation cycle values (an indication of relatively high parasite load), sensitivity by rectal swabs increased to 78.0% (95% CI: 64–89, *P* < 0.0001). These findings suggest that rectal swabs provide less sensitive and reproducible results than bulk stool for the real-time PCR diagnosis of *G. duodenalis.* However, their fair sensitivity for higher parasite loads suggests that swabs may be a useful tool for detecting higher burden infections when stool collection is excessively expensive or logistically challenging.

## INTRODUCTION

The protozoan flagellate *Giardia duodenalis* (syn. *Giardia intestinalis* and *Giardia lamblia*) parasitizes the human small intestine and is the etiological agent of giardiasis, a globally prevalent diarrheal disease. The life cycle of *G. duodenalis* entails a dormant yet infectious cyst phase and a motile, replicating trophozoite phase, with its morphology typified by an adhesive disc significantly implicated in virulence.^1–3^ Genomic analyses suggest that *G. duodenalis* is a species complex comprised of eight distinct groups, assemblages A–H, with assemblages A and B commonly associated with human hosts.^4,5^ Although many infections are asymptomatic, giardiasis manifests with diverse symptomology and may contribute to physical and cognitive stunting in chronic cases.^6^ Children are often at high risk for *G. duodenalis* infection relative to other age-groups.^7–11^

Although microscopic assessment of ova, cysts, and parasites from bulk stool is the current standard of practice for enteric parasite diagnostics,^12–14^ it has been shown to be insensitive and subject to misclassification error relative to molecular methods for both protozoa and helminths.^15–18^ As evidence supports the adoption of molecular pathogen detection in lieu of microscopy, bulk stool remains the recommended specimen type for *G. duodenalis* diagnosis.^19,20^ For some enteropathogens, however, evidence suggests that rectal swabs may provide comparable diagnostic sensitivity with multiple potential benefits. Rectal swabs may offer greater ease of storage and transport in the absence of flammable or toxic preservatives commonly used for stool,^21^ reduced hazardous and biohazardous waste generation during sample collection and processing, and rapid collection.^22^ The latter may be important for short-stay inpatient care, pediatric diagnostics, field settings, or other contexts in which sample collection and diagnostic turnaround must be streamlined. In addition, as rectal swabs are much faster and easier to obtain from young children than bulk stool while remaining minimally invasive, their use could facilitate greater child enrollment in epidemiological studies targeting this at-risk demographic.

The utility of rectal swabs for enteropathogen detection has been explored in the context of bacterial, viral, and eukaryotic disease agents. Statistically insignificant differences in the sensitivity of stool- versus swab-based PCR diagnostics have been observed for *Cryptosporidium*,^23,25^ *Norovirus*,^26,27^ *Rotavirus*,^28^ *Clostridium difficile*,^29^ and commensal bacteria of the gut microbiota.^30,31^ Although PCR sensitivity by rectal swabs may exceed that of bulk stool for *Shigella* and *Campylobacter*, swabs have been seen to underperform in the PCR detection of *G. duodenalis*.^23,24^

To further evaluate the validity of rectal swabs for the molecular detection of *G. duodenalis*, we subjected 136 paired rectal swab and bulk stool specimens from children living in southwest Niger to DNA isolation and real-time PCR (qPCR). Unlike other studies comparing the validity of rectal swab and bulk stool sample collection in clinical populations, this study focused on a population-based representative sample and examined reproducibility by collecting duplicate samples from 50 participants.

## METHODS

### Study population.

This study was nested within a cluster-randomized trial evaluating the effects of azithromycin mass drug administration on child mortality in rural Niger (*Macrolides Oraux pour Réduire les Décès avec un Oeil sur la Résistance* (MORDOR); clinicaltrials.gov NCT02048007).^32^ Study communities were located in Boboye Department, Dosso Region. Within nine of the 30 MORDOR study communities, 447 children aged 0–4 years were randomly selected to participate in the trial, of whom 94 agreed to participate in the rectal swab sub-study.

### Sample collection.

On the day of the study visit, selected children and their accompanying caregivers convened in a centralized location in each community. Trained field examiners collected a rectal swab and a stool specimen from each child. The examiner first cleaned the rectal area with a disinfectant wipe and then inserted a sterile swab (FLOQSwabs™, Copan Diagnostics, Murrieta, CA) of 1–3 cm into the child’s anus and gently turned the swab 180°. The examiner placed the swab into an empty 15 mL sterile tube and put the tube on ice. After swab collection, caregivers were instructed to have their children defecate in a potty chair lined with a black plastic bag. When the caregiver returned the stool to the collection station, the field examiner collected two 0.5 mL specimens of stool and deposited them into two empty 2 mL tubes. The examiner also graded the consistency of the stool sample according to the modified Bristol stool form scale for children (Supplemental Material 1).^33^ Among a random subset of 50 children, field examiners collected a duplicate rectal swab and/or a duplicate aliquot from the bulk stool sample, resulting in 50 duplicates of each specimen type. Both rectal swabs and bulk stool samples were immediately placed on ice and transported to a −20°C freezer by the end of the day. As in prior studies examining rectal swabs as a source of *Giardia* DNA for qPCR diagnosis, rectal swabs were preserved without media,^22,23^ as was bulk stool.

### DNA isolation from paired bulk stool and rectal swab specimens.

#### DNA isolation from bulk stool.

Isolation of total DNA from bulk stool followed a widely used standard operating procedure optimized for the qPCR detection of enteric parasites, modified from the MP Biomedicals FastDNA™ Spin Kit for soil extraction protocol (Supplemental Material 2). All DNA isolations used the MP Biomedicals FastDNA Spin Kit and the MP Biomedicals FastPrep-24™ 5G homogenizer (Santa Ana, CA). Each DNA isolation was spiked with 1 µL of pDMD801 plasmid at a stock concentration of 100 pg/μL, before the addition of a DNA-binding matrix solution containing silica and ceramic. This plasmid, synthesized at Smith College, served as an internal amplification control (IAC) following previously described recommendations.^34^ After isolation, DNA samples were stored at −20°C.

#### DNA isolation from rectal swabs.

Isolation of total DNA from rectal swabs followed the protocol outlined for bulk stool, with the exception of a front-end amendment describing swab preparation and incubation preceding cell lysis (Supplemental Material 2). To develop this amendment, a literature review was performed focusing on existing rectal swab DNA isolation protocols for the detection of enteropathogens.

Before physical and chemical cell lyses, rectal swabs were removed from the 15 mL conical tubes used for transport and storage by grasping the swab stem with clean forceps. The swab stem was trimmed above the swab tip using clean scissors, simultaneously transferring each tip to a 2 mL tube. Subsequently, 1 mL of sodium phosphate buffer was added to each 2 mL tube to serve as an elution buffer for biological material on the swab surface. Based on previously described recommendations, each 2 mL tube was then subjected to an 18-hour incubation of gentle and continuous vortexing at room temperature.^35^ The remainder of the DNA isolation protocol described for bulk stool was then performed on the 1 mL of sodium phosphate buffer used for sample elution, and swab tips were discarded. After isolation, DNA samples were stored at −20°C.

#### Multi-parallel real-time PCR.

DNA isolations were subjected to multi-parallel qPCR for the detection of *G. duodenalis* and for IAC analysis. A previously validated assay targeting the small subunit ribosomal RNA gene was used for the detection of *G. duodenalis*.^36^ Stool-derived and swab-derived DNA extracts were run on separate 96-well plates, with each sample in duplicate 7 μL reactions. This study used TaqPath ProAmp Master Mix (ThermoFisher Scientific, Waltham, MA) and the Applied Biosystems™ StepOnePlus™ Real-Time PCR System (ThermoFisher Scientific, Waltham, MA). Primer oligonucleotides were synthesized by Integrated DNA Technologies, Inc. (Coralville, IA). Fluorescently labeled probes featured a 5′ FAM (fluorescein) fluorophore, an internal ZEN™ quencher, and a 3′ 3IABkFQ quencher (Integrated DNA Technologies, Inc., Coralville, IA). Cycling conditions entailed an initial 2-minute incubation at 50°C, followed by a 10-minute incubation at 95°C and 40 cycles of 1) a 15-second denaturation step at 95°C and 2) a 1-minute annealing and extension step at 60°C for the *G. duodenalis* assay and at 59°C for the IAC assay.

#### PCR data interpretation and quality control.

Samples registering exponential amplification curves with quantitation cycle (C_q_) values less than or equal to 38 in both duplicate wells were evaluated as positive for the respective target.^15^ Samples for which neither independent reaction showed amplification were deemed negative, and samples with discordant results (i.e., one independent reaction amplified, whereas the other did not) were subjected to retesting. In addition, for the IAC assay only, C_q_ values for experimental samples were averaged across the sample set. All samples with duplicate mean C_q_ values greater than two SDs above the sample set mean were assessed as suboptimal in DNA isolation quality. Any samples deemed suboptimal by the IAC assay were eliminated from the dataset.

For the *G. duodenalis* assay, a dilution series of genomic DNA was run as a positive control on each qPCR plate in concentrations of 10 pg/μL, 1 pg/μL, and 100 fg/μL. Each IAC assay plate featured a dilution series of the pDMD801 IAC plasmid in concentrations of 10 pg/μL, 100 fg/μL, and 1 fg/μL. Full-plate retesting was required of any qPCR plate containing positive control duplicates with a mean C_q_ value greater than two SDs above the mean for the respective plasmid concentration.

No-template controls were run in quadruplicate 7 μL reactions on every plate for both assays. Negative controls used 2 μL of molecular biology grade water in place of 2 μL DNA template, and amplification in any of the four wells elicited full-plate retesting.

Technicians performing DNA isolations and qPCR data management were masked to all metadata associated with individual samples, including identifiers linking corresponding rectal swab and stool samples or specimen pair duplicates. During DNA isolation, salient rectal swab characteristics were noted by the technician in a standardized form, including the presence of condensation in the swab storage tube and visible feces on the swab surface.

#### Statistical methods.

We used an unweighted kappa to assess the reliability of molecular detection of *G. duodenalis* across duplicate bulk stool samples and duplicate rectal swabs. Following the precedent of previous studies comparing the PCR sensitivities of multiple specimen types or same-target assays, we considered any individual who tested qPCR positive in at least one specimen replicate by bulk stool or by rectal swab to be a true positive.^23,25,37,38^ These true positives were used as the reference standard to calculate the sensitivity of each collection method. As the collection of human feces by two different methods would have no bearing on the species specificity of a previously validated qPCR assay, this study did not consider effects on diagnostic specificity. Given standard recommendations to collect multiple same-patient samples for maximized accuracy when diagnosing *G. duodenalis*,^20^ aggregate sensitivity when considering both duplicate specimens was also assessed.

To evaluate whether the participant’s age, visible presence of stool on the swab, visible condensation in the swab storage tube, or matched stool consistency affected validity, we included these variables as covariates in multivariate logit models with the dichotomous result of the index test as the dependent variable conditional on the reference standard being positive (i.e., sensitivity).

We evaluated the relationship between rectal swab and bulk stool C_q_ values in a linear regression and compared C_q_ values across paired stool samples, paired rectal swabs, and the first rectal swab with the first stool sample using paired *t*-tests. All analyses were run in R v3.5.2. All code is available on GitHub (San Francisco, CA) (https://github.com/jmaasch).

#### Ethics statement.

Ethical committees from the Niger Ministry of Health and the University of California (San Francisco, CA) granted approval for this study. We obtained verbal informed consent in French from all caregivers.

## RESULTS

### Population characteristics.

Ninety four children participated in the study, with 86 providing paired bulk stool and rectal swab samples. Fifty provided duplicate stool samples, and 50 provided duplicate rectal swabs. The median age was 2 years (interquartile range 1–4), and 58.5% (55/86) of children were female.

#### DNA isolation and real-time PCR quality control.

All qPCR plates met the predefined quality control standards (i.e., C_q_ values of plasmid and gDNA-positive controls were within the defined acceptable range). All DNA isolations passed IAC quality control standards with the exception of two stool samples, which were excluded from further data analysis.

#### Intra-method reliability.

**Figure 1.**
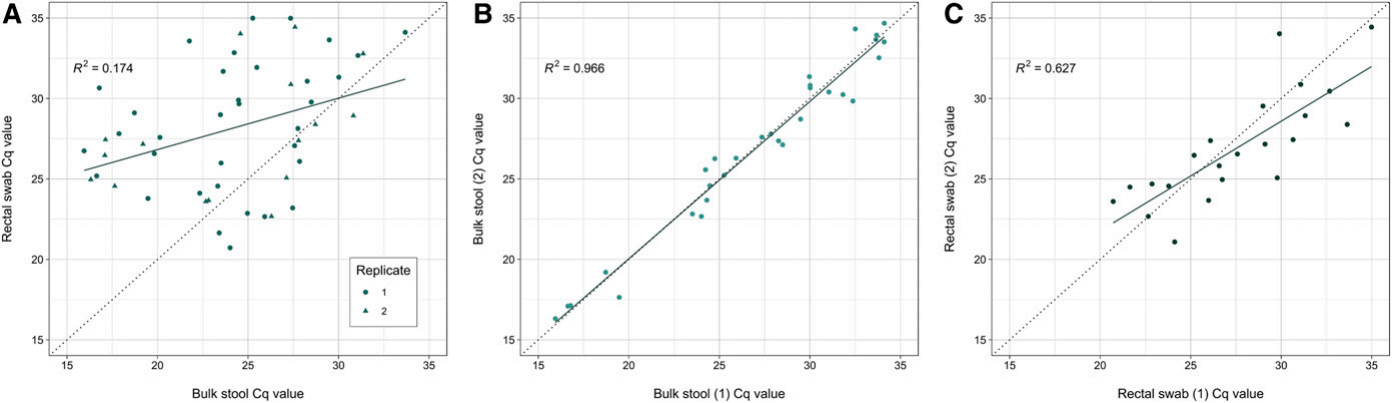
Real-time PCR quantitation cycle (C_q_) value correlations by specimen type (**A**) and sample replicate group (**B** and **C**) for the detection of *Giardia duodenalis*. Plots depict C_q_ value correlations for paired bulk stool and rectal swab specimens (plot A, *N* = 51), bulk stool inter-replicate C_q_ values (plot B, *N* = 31), and rectal swab inter-replicate C_q_ values (plot C, *N* = 22). This figure appears in color at www.ajtmh.org.

The kappa evaluating test agreement was 0.81 (95% CI: 0.54–1.00) among the 49 children with duplicate stool samples and 0.75 (95% CI: 0.47–1.00) among the 48 children with duplicate rectal swabs (Supplemental Table 1). Among the 31 observations with duplicate positive bulk stool samples, the mean C_q_ value was 27.33 (SD = 5.60) in replicate 1 and 27.25 (SD = 5.62) in replicate 2 (*P* = 0.647), with a correlation (*R*^*2*^) of 0.966. For rectal swabs, the mean C_q_ value among the 22 observations with duplicate values was 27.54 (SD = 4.00) in replicate 1 and 26.92 (SD = 3.44) in replicate 2 (*P* = 0.243), with a correlation (*R*^*2*^) of 0.627 (Figure 1).

## METHOD VALIDITY

Of 84 individuals tested, 59 (70.2%) registered positive for *G. duodenalis* infection in at least one specimen replicate by either sample collection method. These positives were classified as true positives for subsequent validity assessments.

*Giardia duodenalis* was detected in 65.5% (55/84) of bulk stool samples and 44.0% (37/84) of rectal swab samples when considering only the first replicate (Table 1). Of the 55 samples positive for *G. duodenalis* by bulk stool, rectal swabs failed to detect 21 (38.2%). Three participants registered positive for *G. duodenalis* infection by rectal swabs and not by bulk stool. Diagnostic sensitivity for the first replicate set was 93% (95% CI: 84–98) by bulk stool and 63% (95% CI: 49–75) by rectal swabs. However, if considering both specimen duplicates to simulate a multi-sample collection scenario, then bulk stool sensitivity was 95% (95% CI: 86–99), whereas rectal swab sensitivity was 68% (95% CI: 54–79). Correlation between paired stool and swab C_q_ values was low (*R*^*2*^ = 0.174) (Figure 1).

**Table 1:** Real-time PCR sensitivity by sample collection method for *Giardia duodenalis* detection

Specimen group	True positive	False positive	True negative	False negative	Sensitivity, % (95% CI)
Either stool sample	56	0	25	3	95 (86–99)
Stool replicate 1	55	0	25	4	93 (84–98)
Stool replicate 2	32	0	14	3	91 (77–98)
Either swab sample	40	0	25	19	68 (54–79)
Swab replicate 1	37	0	25	22	63 (49–75)
Swab replicate 2	25	0	9	14	64 (47–79)

**Figure 2.**
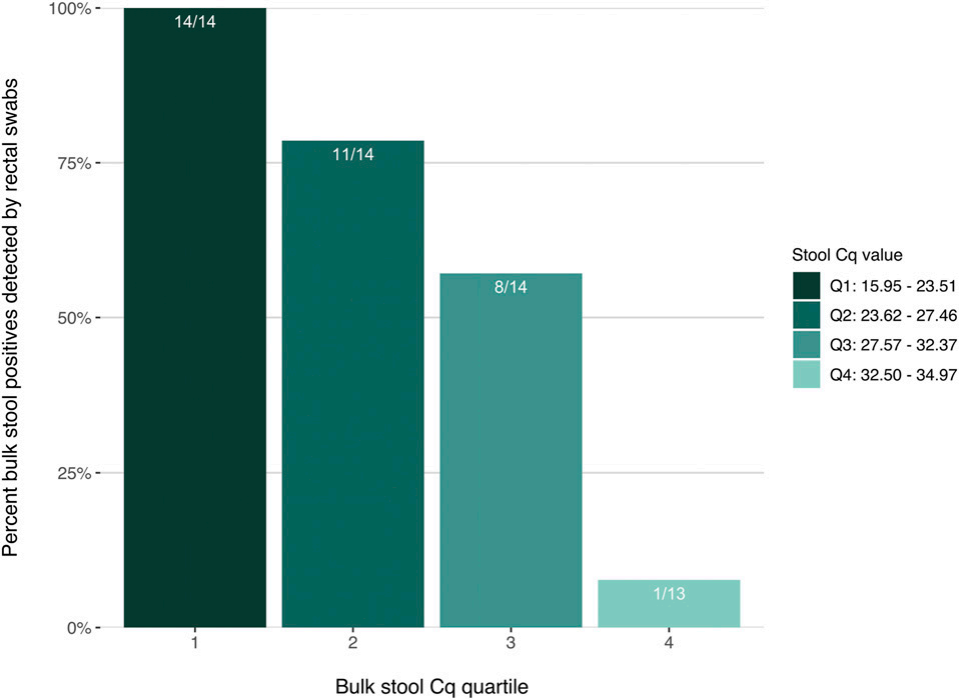
Real-time PCR sensitivity of rectal swab specimens by corresponding bulk stool quantitation cycle (C_q_) value. Rectal swab sensitivity varies when samples are binned by the C_q_ quartile of the participant’s corresponding *Giardia*-positive bulk stool sample (*N* = 55). Data represent the first replicate group of both specimen types. This figure appears in color at www.ajtmh.org.

Binning positive bulk stool results by C_q_ quartile revealed differential rectal swab performance by matched stool C_q_ range (Figure 2). For the first quartile of bulk stool C_q_ values (15.95–23.51), rectal swabs were 100% sensitive (14/14 true positives detected). Within the second and third quartiles (C_q_ 23.62–32.37), rectal swabs were 67.9% sensitive (19/28). Conversely, for bulk stool positives with C_q_ values greater than 32.37 (i.e., samples with the lowest detectable infectious burden), rectal swab sensitivity was only 7.7% (1/13). When restricting to qPCR positives with bulk stool C_q_ values less than or equal to 32.37 (the lowest three quartiles of stool C_q_), rectal swab sensitivity in the first replicate group increased significantly to 78.0% (95% CI: 64–89, *P* < 0.0001).

**Figure 3.**
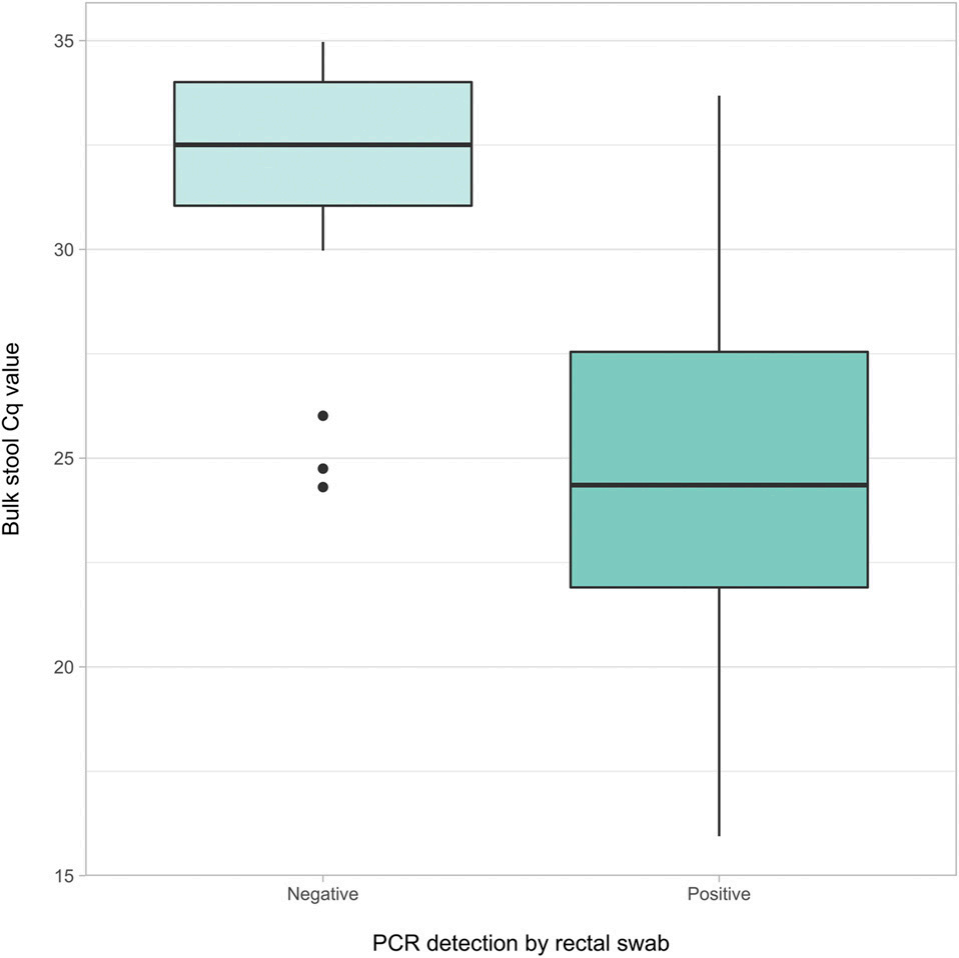
Median quantitation cycle (C_q_) values in bulk stool samples with corresponding qPCR-positive and qPCR-negative rectal swabs. Boxplot depicts median, upper quartile, and lower quartile C_q_ values of bulk stool samples with matched rectal swabs that were positive for *Giardia duodenalis* (*N* = 34) and negative (*N* = 21). Data represent the first replicate group for both specimen types. Outliers were observed among qPCR-negative rectal swabs only (represented by points). This figure appears in color at www.ajtmh.org.

Within the first replicate, the mean C_q_ value of bulk stool samples was 31.79 (SD = 3.17) in the 21 samples with negative paired rectal swabs, compared with 24.26 (SD = 4.33) in the 34 samples with positive paired rectal swabs (*P* < 0.0001) (Figure 3). This notable mean C_q_ difference of 7.53 cycles is equivalent to an approximate 184.8-fold difference in target DNA concentration detected by qPCR, as a C_q_ value increase of 1 corresponds to a doubling of PCR target (assuming 100% efficiency). This indicates that target copy number within the original sample was, on average, 184.8 times greater for bulk stool positives with positive paired rectal swabs than for those with negative paired swabs.

### Effect of covariates on rectal swab validity.

Controlling for the consistency of the matched bulk stool sample, child’s age, visible condensation in the swab storage tube, or the presence of visible feces on the rectal swab surface did not significantly affect diagnostic sensitivity (Supplemental Table 2). Mean C_q_ values were slightly lower for rectal swabs with visible stool (27.5) than rectal swabs without visible stool (28.5); however, the difference was not statistically significant (*P* = 0.54).

## DISCUSSION

### Validity and reproducibiity.

Rectal swabs detected significantly fewer true positives than did bulk stool. Sensitivity was similar to the 62.5% sensitivity observed in a prior study on the qPCR detection of *G. duodenalis* using rectal swab samples.^23^ Sensitivity gains from duplicate specimen testing were greater for rectal swabs than bulk stool. Although intra-test agreement is higher for bulk stool than rectal swabs, these CIs overlap, and rectal swab reliability was fair.

Low fecal mass may explain inferior detection by rectal swabs, as this diminishes the likelihood of a high cyst or trophozoite count in the diagnostic sample. Additionally, the morphology, shedding patterns, or intestinal niche of *G. duodenalis* may not lend themselves to rectal swab detection as well as for protozoan parasites, such as, *Cryptosporidium*, for which rectal swabs have been reported to perform comparably to bulk stool.^23,25^ The intermittent shedding of *G. duodenalis* is the basis for recommendations to collect fecal samples serially and ideally over three separate days,^20,39–41^ as well as for the parasite’s reputation as challenging to diagnose.^19^ In the present study, rectal swab sensitivity did improve through duplicate same-day sample collection. The molecular diagnostic validity of rectal swabs collected on multiple separate days could be an area of further investigation. Furthermore, bead beating has been observed to significantly increase DNA yield from *G. duodenalis* in stool, albeit in a small sample size.^42^ Although the DNA isolation protocol used in the present study did include bead beating, it is possible that there are differential impacts of bead beating on swab samples versus bulk stool.

Although bulk stool is often considered a gold standard specimen type in molecular diagnostics, four individuals deemed positive for *G. duodenalis* infection by rectal swabs were deemed negative in the first bulk stool replicate, with three different positive samples missed in the second stool replicate. Although the present study demonstrates superior detection by bulk stool, it may also suggest that diagnosis by stool is not infallible. This finding may highlight the significance of temporal shedding patterns, heterogeneity of organism dispersal in a single stool specimen, rigorous laboratory and field practices (e.g., stool sample homogenization before aliquoting), and/or multiple sample collection for accurate molecular diagnosis of *G. duodenalis*.

Rectal swab performance may have been impacted by additional aspects of the study design, including the absence of storage media. Although this is not unprecedented among similar studies on *G. duodenalis* diagnosis,^22,23^ the performance of swabs stored with versus without preservative could be interrogated. In a study targeting *C. difficile*, qPCR detection by dry flocked swabs versus bulk stool was 100% concordant, whereas flocked swabs stored in liquid transport medium had significantly lower sensitivity.^43^ However, the referenced study simulated swab collection by dipping swabs in bulk stool samples.

#### Effect of covariates on rectal swab validity.

Clinical diagnostic guidelines generally assume a positive relationship between visible soiling with fecal matter and the diagnostic utility of rectal swabs.^44^ However, recent interrogations of this relationship have concluded that testing rectal swabs that lack visible feces is worthwhile for the PCR detection of common enteric bacteria and viruses.^44,45^ Our results further corroborate this conclusion in the context of a protozoan diagnostic, as we observed no difference in rectal swab sensitivity when controlling for visible feces. There was an indication that C_q_ values were slightly lower in rectal swabs with visible stool, although the difference was not statistically significant. However, we were likely underpowered to evaluate this effect with 24 of 86 rectal swabs having visible stool.

Whereas condensation was hypothesized to encourage enzymatic activity in media-less storage tubes, and thus to negatively affect diagnostic sensitivity, we found that visible moisture within media-less storage tubes did not significantly affect the qPCR detection of *G. duodenalis* by rectal swabs. However, with just 12 of 84 samples featuring visible condensation, we were underpowered to evaluate the effect of condensation on diagnostic sensitivity.

#### Rectal swab sensitivity by parasite load.

The C_q_ value of DNA derived from bulk stool may be considered a proxy for relative target DNA concentration and, thus, for relative pathogen load. By extension, if C_q_ values for matched swab and stool specimens correlate strongly, then qPCR results for rectal swab samples may also be used to describe relative pathogen loads. Furthermore, if rectal swabs perform differently depending on bulk stool C_q_ value, then this may speak to the utility of rectal swabs under different infection intensities.

Mean C_q_ values for bulk stool and rectal swabs differed significantly, and a small proportion of variation in the rectal swab C_q_ value could be explained by the C_q_ value of the paired bulk stool sample, similar to a prior study.^24^ In addition, the C_q_ values of rectal swab duplicates showed a less strong relationship than did stool duplicates. These findings suggest that it may be more challenging to draw conclusions about the relative pathogen load of *G. duodenalis* when performing qPCR on swab-derived DNA versus stool-derived DNA.

The sensitivity of rectal swabs increased substantially when restricting by paired stool samples with low C_q_ values, an indicator of relatively high parasite burden. This phenomenon has previously been observed for *G. duodenalis*, along with several viral and bacterial targets.^24^ The weak performance of rectal swabs in lower pathogen load scenarios may be problematic in population-based studies and monitoring programs, where lower level infections may go undetected while still contributing to morbidity, parasite transmission, and recrudescence.^46–49^ Conversely, this may be an advantage for studies interested in evaluating the morbidity effects of higher burden infections on growth, cognitive stunting, or other significant indicators in childhood development. Using rectal swabs to detect higher burden infections may also be useful for studies assessing intervention effects targeted at higher burden individuals.

Whether the high-C_q_ true positives missed by rectal swabs represent subclinical infections or nonpathogenic colonization could be an area of further investigation. It has been suggested that this may be the case, as organisms which adhere to mucosae and thus may be pathogenic are more likely to be detected by swabs than are organisms within the intestinal lumen, which may be abundant in stool.^22^

## CONCLUSION

Molecular diagnostics for enteric parasites like *G. duodenalis* are increasingly common and typically require bulk stool specimens. Relative to bulk stool, rectal swabs may offer greater ease of collection, storage, transport, and waste disposal. However, the significant loss of diagnostic sensitivity as documented in this study could lead to underestimation of *G. duodenalis* prevalence when using rectal swabs. Given the differential performance of swabs for higher versus lower level infections, this would be problematic in contexts where low-burden infections are common. Poor detection of lower level infections may also be detrimental in control and elimination scenarios, where undetected low-burden infections may still contribute to parasite transmission and recrudescence. Conversely, the fair sensitivity of rectal swabs for diagnosing higher level infections may indicate the utility of swabs as a frontline sampling method in contexts where high-burden infections are most relevant, particularly when bulk stool collection is logistically or financially infeasible.

## Supplemental materials figure and tables

Supplemental materials
