# Supplementary material for: Rectal Swabs as an Alternative Sample Collection Method to Bulk Stool for the Real-Time PCR Detection of Giardia duodenalis
Source: Am J Trop Med Hyg. 2020 Jun 8;103(3):1276–82. doi: 10.4269/ajtmh.19-0909 (PMC7470573; doi:10.4269/ajtmh.19-0909)
Supplement: Supplementary file 1 [file tpmd190909.SD1.doc]

Modified Bristol Stool Form Scale for Children (mBSFS-C)

Translated into French

|  |  | **Original mBSFS-C** | **French** | **Back-translated to English** |
| --- | --- | --- | --- | --- |
| **Type 1** | 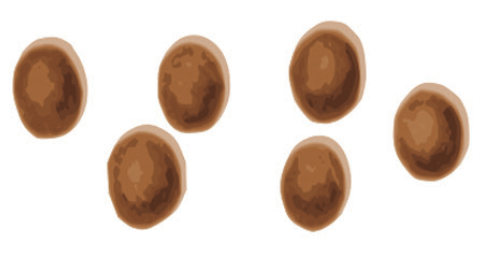 | Separate hard lumps, like nuts (hard to pass) | Petites boules dures et detachées (difficile a evacuer) | Very dry stool (small and round like sheep feces), hard to pass |
| **Type 2** | 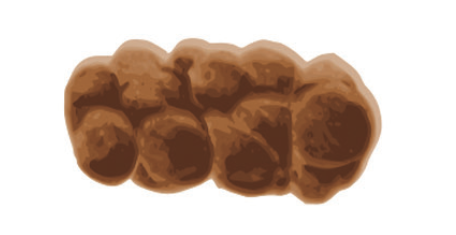 | Sausage shapes but lumpy | Forme d’une saucisse dure et grumuleuse | Dry stool (a single mass of small round feces, like sheep feces formed together) |
| **Type 3** | 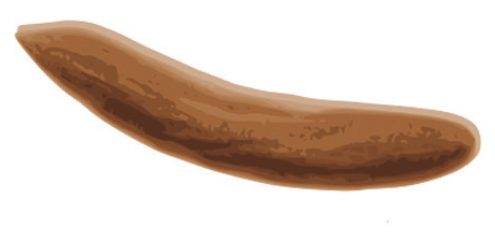 | Like a sausage or snake, smooth and soft | Comme une saucisse ou un serpent, lisse et molle | Soft, not dry and its shape is like snake |
| **Type 4** | 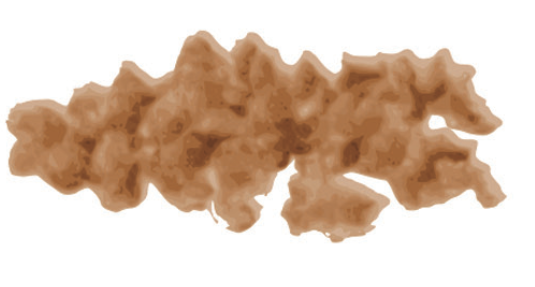 | Fluffy pieces with ragged edges, a mushy stool | Petit fragment, duveteux aux bords irreguliers, selles détrempées | Very soft and irregular shaped |
| **Type 5** | 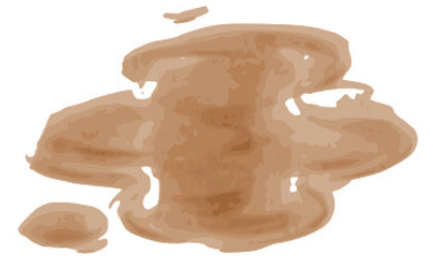 | Watery, no solid pieces | Entierement liquides, aucun fragment solide | Watery stool |
