# Supplementary material for: Rectal Swabs as an Alternative Sample Collection Method to Bulk Stool for the Real-Time PCR Detection of Giardia duodenalis
Source: Am J Trop Med Hyg. 2020 Jun 8;103(3):1276–82. doi: 10.4269/ajtmh.19-0909 (PMC7470573; doi:10.4269/ajtmh.19-0909)
Supplement: Supplementary file 2 [file tpmd190909.SD2.doc]

**Supplemental Table 1. Real-time PCR agreement tables for molecular detection of *Giardia duodenalis* by rectal swabs versus bulk stool**

| ***A*** |  |  |  |
| --- | --- | --- | --- |
|  | **Stool –** | **Stool +** | **Total** |
| **Swab –** | 25 | 19 | 44 |
| **Swab +** | 3 | 37 | 40 |
| **Total** | 28 | 56 | 84 |
|  |  |  |  |
| ***B*** |  |  |  |
|  | **Stool 1 –** | **Stool 1 +** | **Total** |
| **Stool 2 –** | 14 | 1 | 15 |
| **Stool 2 +** | 3 | 31 | 34 |
| **Total** | 17 | 32 | 49 |
|  |  |  |  |
| ***C*** |  |  |  |
|  | **Swab 1 –** | **Swab 1 +** | **Total** |
| **Swab 2 –** | 20 | 3 | 23 |
| **Swab 2 +** | 3 | 22 | 25 |
| **Total** | 23 | 25 | 48 |

Caption: A) Detection by either replicate group. B) *κ* = 0.81 (95% CI 0.54–1.00). C) *κ* = 0.75 (95% CI 0.47–1.00).

**Supplemental Table 2. Covariate effects evaluated for the first rectal swab replicate group relative to true *Giardia duodenalis* positives**

|  | ***N* children** | | **Swab sensitivity (%, 95% CI)** | **Covariate *p*-value** |
| --- | --- | --- | --- | --- |
| Total | | 84 | 63 (49–75) |  |
| Child less than 12 months old | | 11 | 67 (22–96) | 0.89 |
| Feces visible on swab surface | | 15 | 59 (33–82) | 0.64 |
| Condensation visible in storage tube | | 9 | 63 (49–75) | 0.50 |
| Loose or watery texture of matched stool | | 11 | 83 (36–100) | 0.36 |
|  | |  |  |  |
