# Supplementary material for: Rectal Swabs as an Alternative Sample Collection Method to Bulk Stool for the Real-Time PCR Detection of Giardia duodenalis
Source: Am J Trop Med Hyg. 2020 Jun 8;103(3):1276–82. doi: 10.4269/ajtmh.19-0909 (PMC7470573; doi:10.4269/ajtmh.19-0909)
Supplement: Supplementary file 3 [file tpmd190909.SD3.pdf]

SOP_DNA-isolation_rectal-swabs.docx	**DNA ISOLATION FROM RECTAL SWABS**	SMITH COLLEGE

STANDARD OPERATING PROCEDURE (SOP)

ISOLATION OF DNA FROM RECTAL SWABS
FOR MOLECULAR DETECTION OF ENTERIC PARASITES

**SOP AUTHORS**	**Jacqueline R. M. A. Maasch**, Research Associate, Williams Laboratory, Smith College, Northampton, MA, USA<br>**Dr. Rojelio Mejia**, Assistant Professor of Pediatrics and Medicine, Baylor College of Medicine Houston, TX, US
**PRINCIPAL INVESTIGATORS**	Dr. Jeremy Keenan (UCSF), Dr. Kristen Aiemjoy (Stanford), Dr. Steven Williams (Smith College)
**PROJECT LEAD**	Jacqueline R. M. A. Maasch
**SOP VERSION**	1
**LAST UPDATED**	March 2019

If using this SOP, please cite:

Maasch J, Arzika AM, Cook C, Lebas E, Pilotte N, Grant JR, Williams SA, Keenan J, Lietman TM, Aiemjoy K, 2020. Rectal swabs as an alternative sample collection method to bulk stool for the real-time PCR detection of *Giardia duodenalis*. *American Journal of Tropical Medicine and Hygiene. doi:10.4269/ajtmh.19-0909*

SOP development supported by:
UCSF Catalyst Award
Bill & Melinda Gates Foundation
The Blakeslee Fund for Genetics Research at Smith College

INTRODUCTION

This document outlines a procedure for the isolation of total DNA from rectal swabs for the molecular detection of enteric parasites. This procedure is adapted from the MP Biomedicals FastDNA™ Spin Kit for Soil Extraction Protocol and from modifications to the manufacturer's protocol made by Dr. Rojelio Mejia (2014). Sample preparation and incubation stages of this protocol were developed by Jacqueline Maasch (2019), while the remainder of the SOP is performed in accordance with that developed by Mejia (2014).

Validation of this procedure subjected rectal swabs collected in Niger to DNA isolation and quantitative polymerase chain reaction (qPCR) diagnostics for *Giardia duodenalis* (Maasch et al 2020). **Note: This procedure takes two days to perform. An 18-hour (overnight) incubation is required.** The duration of this incubation period was selected based on findings by Adamowicz et al (2014).

SAMPLE MATERIAL

Rectal swabs. Validated using swabs without preservative only.

LITERATURE

- MP Biomedicals FastDNA™ Spin Kit for Soil Extraction Protocol.
- Mejia, R. 2014. Standard operating procedure for isolation of DNA from human stool samples.
- Maasch J, Arzika AM, Cook C, Lebas E, Pilotte N, Grant JR, Williams SA, Keenan J, Lietman TM, Aiemjoy K. 2020. Rectal swabs as an alternative sample collection method to bulk stool for the real-time PCR detection of *Giardia duodenalis. Am. J. Trop. Med. Hyg.*
- Adamowicz MS, Stasulli DM, Sobestanovich EM, Bille TW. Evaluation of methods to improve the extraction and recovery of DNA from cotton swabs for forensic analysis. *PLOS ONE 9*(12).
- Deer DM, Lampel KA, González-Escalona N. 2010. A versatile internal control for use as DNA in real-time PCR and as RNA in real-time reverse transcription PCR assays. *Letters in Applied Microbiology 50*:366–372.

EQUIPMENT & REAGENTS

MP Biomedicals FastDNA™ Spin Kit for Soil:

- Lysing Matrix E (50 x 2.0 mL tube)
- Sodium Phosphate Buffer (1 x 60 mL)
- MT Buffer (1 x 8 mL vial)
- PPS Solution (1 x 25 mL bottle)
- Binding Matrix (2 x 30 mL bottle)
- SPIN Modules, i.e. Spin Filters (50)
- Catch / Collection tubes (50)
- Concentrated SEWS-M (1 x 12 ml bottle)
- DES (1 x 20 ml bottle)
- BBS Gel Loading Dye (not used for this procedure)

Required materials not included in kit:

- Micropipettors and tips (p1000, p200, p20, p10): We recommend:
  - 1-10 µL Gilson Single Channel Pipette (VWR # 76178-350)
  - 2-20 µL Gilson Single Channel Pipette (VWR # 76178-352)
  - 20-200 µL Gilson Single Channel Pipette (VWR # 76178-052)
  - 100-1000 µL Gilson Single Channel Pipette (VWR # 76178-054)

- pDMD801 plasmid control (*see Deer et al 2010*)
- 2.0 mL round-bottom microcentrifuge tubes (USA Scientific # 1620-2700)
- 1.7 mL microcentrifuge tubes (Phenix Research Products # MAX-715)
- Permanent marker
- Microcentrifuge tube openers (USA Scientific # 1400-1508)
- Microcentrifuge tube racks
- Waste beaker
- Microcentrifuge
- Vortex
- Vortex adapter for microcentrifuge tubes (accommodating at least 24 tubes)
- Parafilm
- Microcentrifuge tube storage boxes
- Sterile loops (Fisher Scientific # 22-363-605)
- Sterile razor blades or scissors
- Disposal container for sharp objects (Sharps container)
- Kimwipes or equivalent
- Bleach or equivalent laboratory disinfectant
- MP Biomedicals FastPrep-24™5G Instrument with metal sample holder (MP Biomedicals # 116005500)

PROCEDURE

PRE-PROCEDURE REAGENT PREPARATION

1. Prepare SEWS-M Wash Solution: Prior to use, 100 mL of 100% Ethanol must be added to the 12 mL of concentrated SEWS-M Wash Solution which is provided with the kit. To add ethanol, use a clean graduated cylinder and rinse the graduated cylinder with ethanol before use.

CLEANING WORK STATIONS

1. Prior to beginning, clean all equipment and surfaces to be used during the procedure with freshly prepared 10% bleach. Use ultrapure Milli-Q water to dilute bleach.

SAMPLE PREPARATION & INCUBATION: DAYS 1 & 2 (Maasch 2019)

1. Remove any parafilm from 15 mL conical tube containing rectal swabs.
2. Clean the exterior surfaces of all 15 mL tubes with 10% bleach.
3. Prepare decontamination set-up to facilitate hygienic transfer of rectal swabs from 15 mL conical tubes to 1.7 mL tubes.

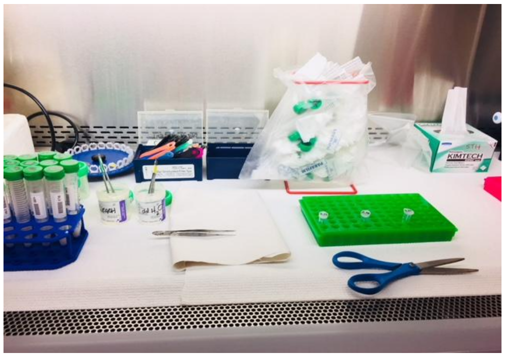

   a. Prepare small containers of 1) 10% bleach / 90% MilliQ water and 2) pure MilliQ water.
   b. Assemble Kim Wipes, multiple tweezers, razor blade, parafilm, scissors, and paper towel.
4. Lay out 1.7 mL tubes in tube rack and label with unique sample identifiers.
5. Transfer swab from 15 mL conical tube to 1.7 mL tube.
   a. Remove swab from conical tube by grasping the stem with clean tweezers.
   b. Using clean scissors, clip swab stem above swab tip, simultaneously transferring to 1.7 mL tube.
   c. Between samples, soak tweezers and scissors in bleach, rinse in MilliQ water bath, and dry on paper towel and/or with Kim Wipes.
6. Add 1 mL Sodium Phosphate Buffer to 1.7 mL tube containing swab tip.
   a. Use the same tip to load all tubes unless tip touches sample tube.
7. Parafilm 1.7 mL tube to prevent spills during incubation.
8. **Vortex gently and continuously for 18 hours.**

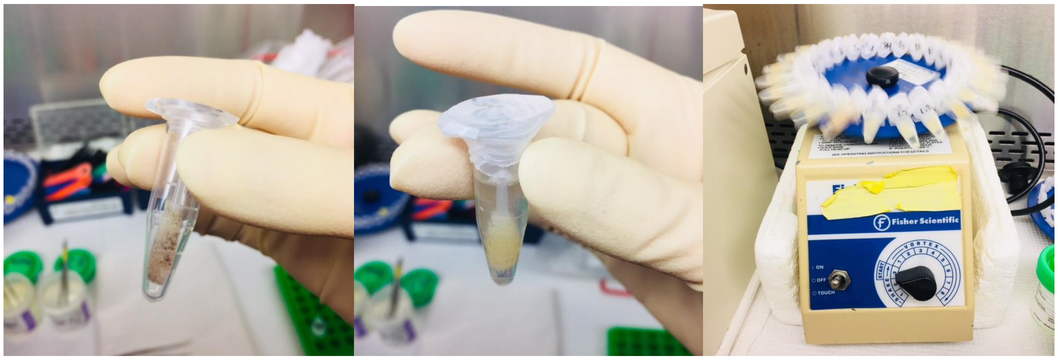

9. Lay out MP Bio lysis tubes in tube rack and label with unique sample identifiers.
10. Add 122 µL MT Buffer to lysis tubes.
    a. Use the same tip to load all sample tubes.
11. Remove 1.7 mL swab elution tubes from vortex platform and place in tube rack.
12. Use razor blade to carefully remove parafilm from 1.7 mL swab elution tubes.
13. Pulse-spin swab elutions for 3 seconds in centrifuge to clear elution from tube lid.
14. Open each swab elution tube with a unique, clean tube opener.
15. Transfer 900 µL Sodium Phosphate swab elution to lysis tubes containing MT Buffer and discard swab.
    a. While tubes are open, place caps face down on paper towel so that caps are facing up.

DNA ISOLATION (Mejia 2014)

16. Homogenize the samples for 40 sec using the FastPrep Instrument on a speed setting of 6.0 (can use the Alpowa wheat tissue setting).
17. Centrifuge tubes at 14,000 x g for 10 min.
18. While centrifuging, for each sample, add 250 µl of PPS to a new 2 ml tube
    a. Use the same tip to load all samples.
19. For each sample, following centrifugation, pipet the supernatant from Step 5 into a new 2 ml tube prepared in Step 6 and invert the tube 10 times by hand to mix.
20. Centrifuge at 14,000 x g for 10 min.
21. While centrifuging, for each sample, add 500 µl of Binding matrix into each of two new 1.7 ml tubes.
    a. Prior to pipetting, vortex to resuspend the binding matrix vigorously and pipette matrix from the bottom of the bottle.
    b. Use the same tip to load the binding matrix into all tubes.
    c. To ensure that matrix remains mixed, vortex binding matrix after every 3–5 tubes aliquoted.
22. Add 1.0 µl of internal control (pDMD801 plasmid, 100pg/µl) to each sample tube spun in step 10.
    a. **Do not MIX following the addition of the control.**
    b. Open sample tubes with a tube opener.
23. For each sample, add approximately 550 µL of the supernatant from step 12 to each binding matrix-containing tube from Step 11.
    a. Note: Sample volumes may vary. Divide the supernatant equally between the two binding matrix-containing tubes.
    b. Use the same tip for each sample, but change tips between samples.
24. Slowly hand rock all tubes for 2 min.
    a. Note: Samples can be store at 4 °C for 1 hour after this step.
25. Let all sample-containing tubes sit at room temperature for 5 min.
26. Remove nearly the entire volume of supernatant from each tube.
    a. Remove supernatant at an angle, being careful not to disrupt the matrix
    b. Remove enough supernatant to leave approximately 1 mm of volume behind in each tube. Dispose of the supernatant in a waste container.
    c. Use the same tip for each sample, but change tips between samples.
27. Remove the entire volume of binding matrix from each tube and immediately add this volume to a spin filter with collection tube. At this point, the matrix from both tubes containing a single sample can be recombined into a single spin filter with collection tube.
    a. For each sample, use the same tip that was used during Step 16.
    b. Gently pipette up and down to re-suspend the matrix before transferring it to the spin filter with collection tube.
28. Centrifuge all samples at 14,000 x g for 2 min.
    a. Depending on the consistency of the sample, more spins might become necessary to fully elute fluids from the spin filter.
29. Discard the liquids from each tube.
    a. Tap tube on a clean paper towel after dumping the liquids. Make sure to tap each tube in a new location and leave plenty of space between where tubes are tapped.

30. Add 500 µl of PREPARED SEWS-M to the matrix obtained from each sample and mix samples with gentle pipetting.
    a. Note: Take care not to pierce the membrane at the bottom of the filter tube.
31. Centrifuge all samples at 14,000 x g for 2 min.
32. Empty the collection tube and discard the fluid.
33. Centrifuge all samples at 14,000 x g for 2 min.
    a. Note: This additional centrifugation step helps to dry the matrix.
34. Replace the tube with the final collection tube.
35. At room temperature, air dry all filters for 5 min.
    a. Leave tube lids open while drying.
36. Add 100 µl of DES to each sample tube and mix matrix with gentle stirring.
    a. Add DES very slowly directly on top of the matrix.
    b. Do not pipette up and down. Just stir with your pipet tip.
    c. Do not vortex.
37. Centrifuge all samples at 14,000 x g for 2 min to recover DNA.
38. For short-term storage (<1 week), place samples at 4 °C. For long term storage (>1 week), store samples at -20 °C.
